# Compensation for the source drift of a free-electron laser beamline by adjusting the fixed-focus constant of the grating monochromator

**DOI:** 10.1107/S1600577524012116

**Published:** 2025-02-03

**Authors:** Chaofan Xue, Lian Xue, Yong Wang, Renzhong Tai

**Affiliations:** a Shanghai Advanced Research Institute Chinese Academy of Sciences Shanghai201210 People’s Republic of China; b Shanghai Synchrotron Radiation Facility, Shanghai201204, People’s Republic of China; RIKEN SPring-8 Center, Japan

**Keywords:** free-electron laser facility, longitudinal source point drift, energy-resolving power, *C*_ff_ value compensation

## Abstract

For large-scale free-electron laser facilities based on linear accelerators, the laser saturation point is not frequently fixed in the undulator, which will cause a longitudinal source point drift of the beamline. The longitudinal source point drift will cause an instability in the performance of the beamline, especially affecting the energy-resolving power of variable-line-spacing grating monochromators for soft X-ray beamlines. A method of adjusting the fixed-focus constant to compensate for this longitudinal source point drift is introduced in this work. Simulation results indicate that this method can effectively recover the energy-resolving power of the grating monochromator.

## Abstract 2

A method for compensating for the source drift of a free-electron laser beamline by adjusting the *C*_ff_ value of a grating monochromator is introduced.

## 1. Introduction

Due to many unique properties, such as transversely coherent and ultrashort-pulsed sources, high brightness and peak power, tunable wavelengths and wide spectral range (Saldin *et al.*, 1995 ▸; Tiedtke *et al.*, 2004 ▸; Amann *et al.*, 2012 ▸; Togashi *et al.*, 2013 ▸; McNeil & Thompson, 2010 ▸), electron linac accelerator-based free-electron laser (FEL) sources have been widely used in a variety of basic scientific research fields, including condensed matter physics, advanced materials and surface physics, atomic and molecular physics, chemistry and biology. Driven by scientific requirements, several FEL facilities (Ackermann *et al.*, 2007 ▸; Emma *et al.*, 2010 ▸; Ishikawa *et al.*, 2012 ▸; Allaria *et al.*, 2012 ▸; Kang *et al.*, 2017 ▸; Prat *et al.*, 2020 ▸; Decking *et al.*, 2020 ▸), from low repetition rate to high repetition rate, have been built or are under construction worldwide to meet the needs of scientists for ultra-high-brightness and ultra-short-pulse sources.

In a FEL facility, several or dozens of undulators are frequently required to saturate the FEL. This means that the electron beam needs to travel several tens or hundreds of meters after entering the undulator. Generally speaking, the saturation point of the FEL serves as the source point of the beamline because the beam size and divergence angle no longer change intensely after the FEL reaches saturation. Regrettably, the saturation point positions in the undulator section do not remain at the same position for different photon energies. The different saturation point’s position means a variable source point position for the beamline, which is not good news for the scientific instruments because a variable source point position will affect the focusing spot and energy resolution. To compensate for an unfixed source point position, both accelerators and beamlines can provide their own solutions. From the accelerator side, a feasible solution is that the gap of upstream undulators can be opened to keep the saturation point of the laser in a designed position for the beamline, which requires several or more undulators to move simultaneously. For different photon energies, there is a variation in the number of undulators that need to be opened. High requirements have been put forward for mechanical motion, motion control and parameter adjustment of the accelerator. From the beamline side, if the photon beam is directly focused by the Kirkpatrick–Baez (KB) mirrors without passing through the monochromator, the longitudinal source point drift can be compensated to a certain extent through the bending mechanism of the KB system. However, compensating for longitudinal source point drift through a bending mechanism is a challenge for the monochromator.

In this work, a method of compensating for longitudinal source point drift by adjusting the parameters of the grating monochromator is introduced. By fine-tuning the fixed-focus constant (*C*_ff_) of the grating, compensation for longitudinal source point drift can be effectively achieved, while maintaining the energy-resolving power of the grating monochromator.

## 2. Optimization method

In a variable-included-angle plane-grating monochromator, a plane mirror is used in front of the diffraction grating to provide the variable-included-angle capability (Petersen, 1982 ▸). The fixed-focus constant (the so-called *C*_ff_ value) is the core parameter of a grating monochromator and is defined as
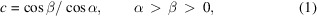
where α is the angle of normal incidence in the grating and β is the angle of diffraction. The focal distance is at a fixed distance if *C*_ff_ is kept constant. When the non-parallel illumination condition on the grating is replaced by the parallel illumination condition, the *C*_ff_ value can be changed without moving the exit slit (Follath & Senf, 1997 ▸). Therefore, the monochromator could be optimized for grating efficiency or rejection of harmonics. Due to its wide energy coverage range and flexible operation mode, the variable-included-angle plane-grating monochromator (VIA-PGM) is widely used in soft X-ray beamlines (Flechsig *et al.*, 2004 ▸; Warwick *et al.*, 2004 ▸; Xue *et al.*, 2010 ▸). Regretfully, a concave mirror is required downstream of the grating to focus the dispersed beam at the exit slit for both illumination conditions. These re-focusing mirrors will increase the aberration of the optical system and lead to a decrease in the energy-resolving power of the PGM. With the development of grating processing technology, variable-line-spacing (VLS) gratings are being increasingly adopted in the PGM design due to their aberration correction function (Reininger & Castro, 2005 ▸; Xue *et al.*, 2014 ▸; Xue *et al.*, 2019 ▸).

For a VLS grating, the line spacing *d* is a function of position *w* in the dispersive direction. The function can be expanded as a power series of *w*, namely
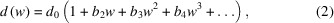
where *d*_0_ is the line spacing at the center of the grating, and *b*_2_, *b*_3_ and *b*_4_ are the space-variation parameters. The defocus term (*F*_20_) and the coma term (*F*_30_) in an optical path function can be eliminated by choosing an appropriate linear coefficient term *b*_2_ and quadratic term *b*_3_, respectively, according to
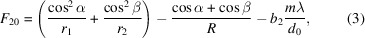

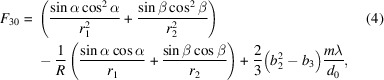
where *m* is the diffraction order, α is the incidence angle, β is the diffraction angle, *r*_1_ is the objective distance, *r*_2_ is the imaging distance and *R* is the grating radius (for a plane grating, *R* → ∞). When *F*_20_ = 0, the focusing condition is satisfied.

Usually, the operating conditions for an optimized grating will not be changed because the grating parameters have already been determined and can no longer be changed once the processing is finished. On the other hand, this means that the grating can no longer function properly under the designed parameters when the operating conditions change. Starting from the focusing equation and taking a plane grating as an example, we attempt to find a compensation method that can guarantee the performance of the grating when the operating conditions change. Moving the exit slit in a beamline is not an optimal solution because the source position of the downstream focusing mirrors will change. Once the grating processing is finished, the *b*_2_ coefficient of the grating will be determined and can no longer be altered, as previously mentioned. Fortunately, for an incident beam at a certain wavelength under a fixed diffraction order, it is possible to compensate for the changes in the source point by adjusting the incident angle of the grating. It is essential that the incident angle of the grating at this time meets both the grating equation and the focusing equation simultaneously. Therefore, by combining the grating equation with the focusing equation, we can obtain
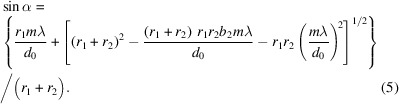
This means that, for an incident beam at a certain wavelength under a fixed diffraction order, when the image distance and the *b*_2_ coefficient of the grating are determined, different grating distances correspond to different grating incidence angles. The change in grating object distance can be compensated by adjusting the *C*_ff_ value of the grating.

**Figure 1:**
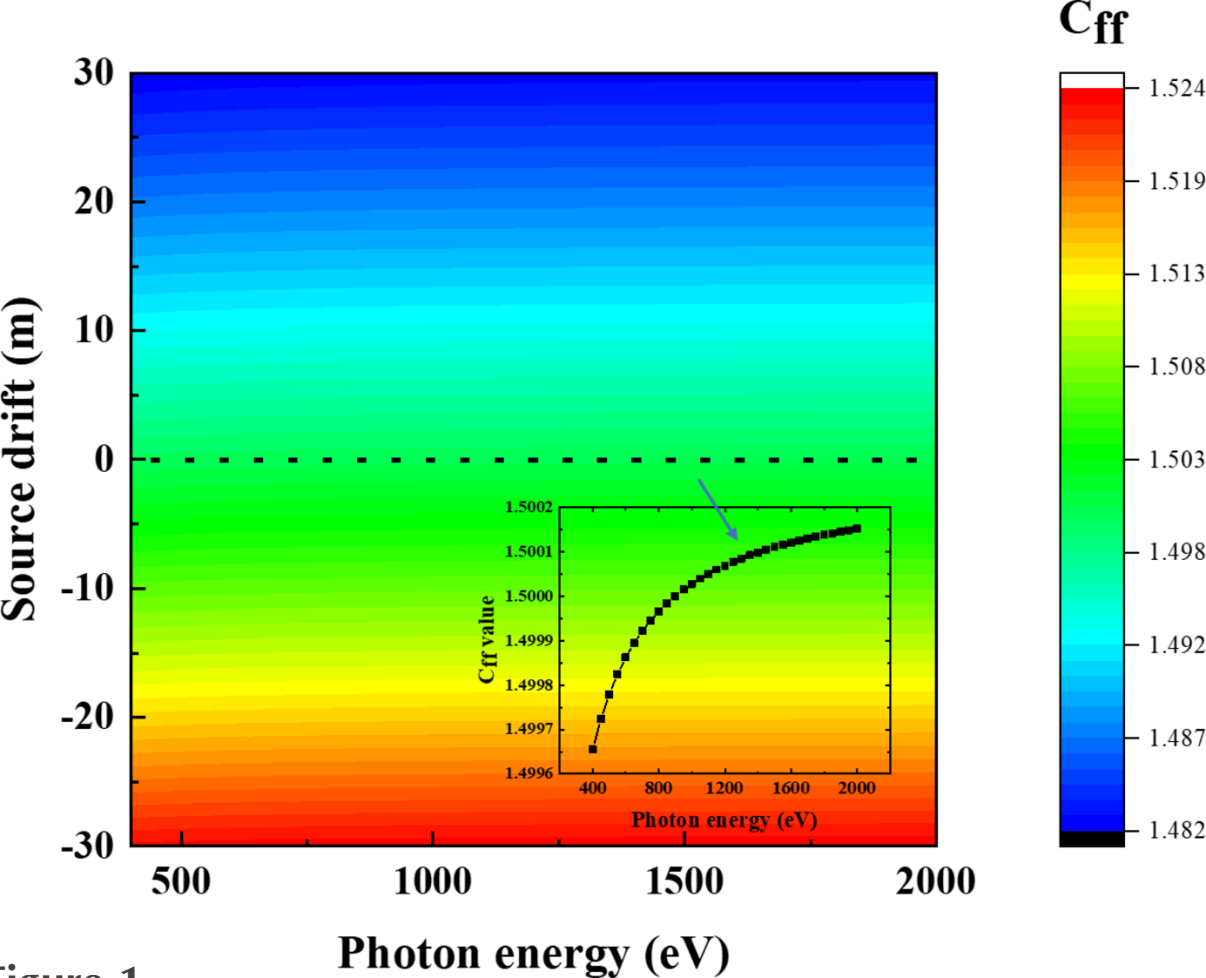
Compensated *C*_ff_ value under different source drift levels. The dashed line represents the initial designed *C*_ff_ value as shown in the insert figure.

A 200 lines mm^−1^ grating is taken as an example to verify the feasibility of the compensation method. The object distance and imaging distance of the grating are 200 m and 95 m, respectively. The initial designed *C*_ff_ value is 1.5 optimized at 900 eV. The basic parameters of the grating are summarized in Table 1 ▸. The *C*_ff_ values of the compensated grating operation are shown in Fig. 1 ▸, considering the different levels of source drift. In the case of a source drift of ±30 m (±15% of the designed grating object distance), the change in *C*_ff_ value is only less than ±3%. Such a small *C*_ff_ value change can be achieved for a PGM, making it feasible to compensate for the source point drift by adjusting the *C*_ff_ value of the PGM.

**Figure 2:**
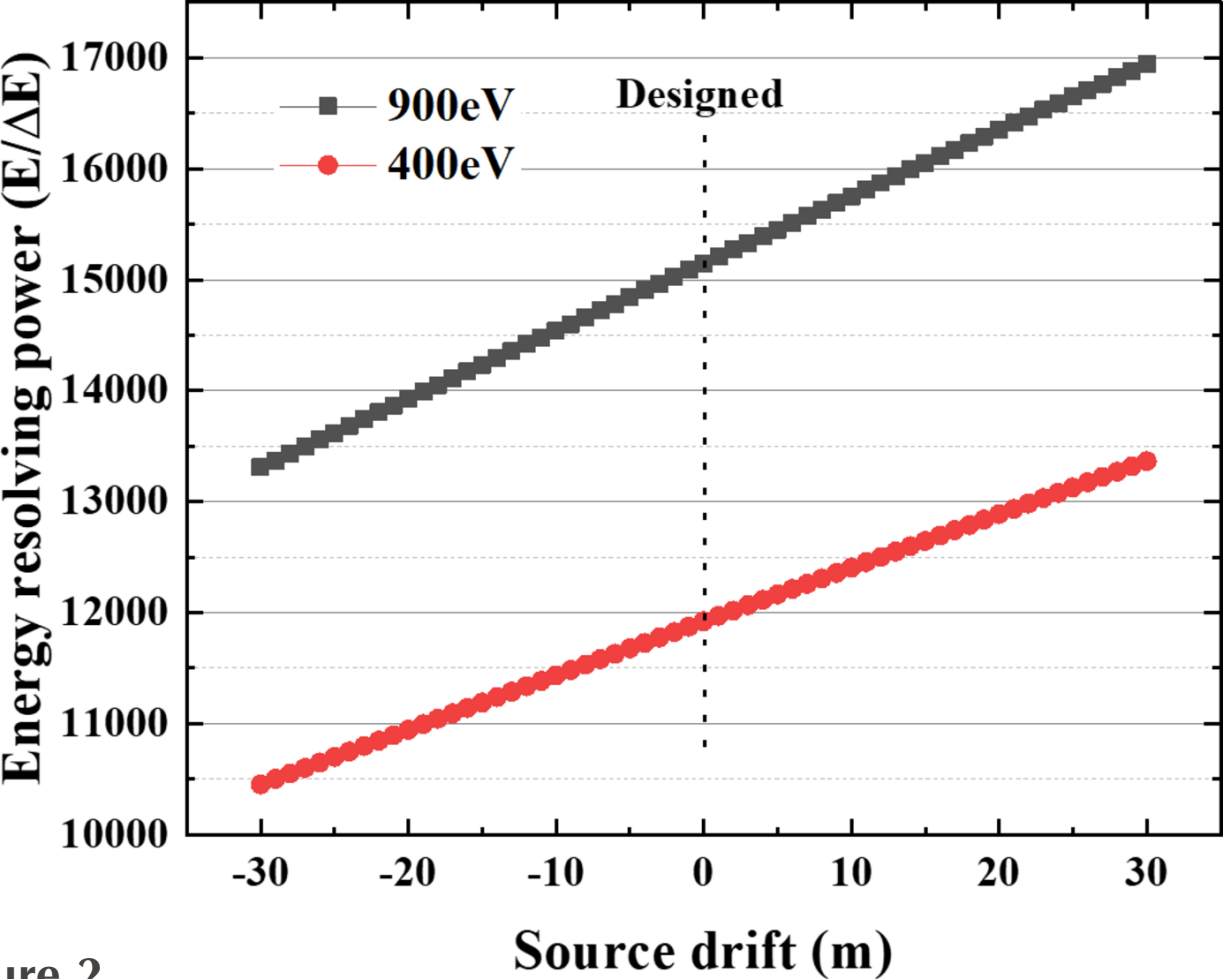
Compensated energy-resolving power of the grating monochromator under different source drift conditions

Compensating for *C*_ff_ values is the key to regaining the energy-resolving power of the grating monochromator. Fig. 2 ▸ shows the energy-resolving power of the grating monochromator after compensating for *C*_ff_ values under different source drift conditions. The energy-resolving power calculated in this study is mainly determined by six factors: exit slit size, meridian slope errors of the grating and pre-mirror, aberrations from the defocus and the coma, and the grating diffraction limit. High-order aberrations (smaller than *F*_30_) are small and negligible. The energy-resolving power is defined as the inverse of the energy resolution. Their contributions to the energy resolution Δ*E* are as follows,

exit slit size: 
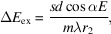


meridian slope errors of grating: 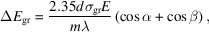


meridian slope errors of mirror: 
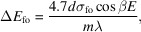


aberration from defocus: 
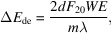


aberration from coma: 
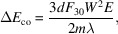


diffraction limit: 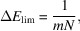
and 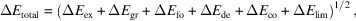
where*s* is the exit slit size, σ_gr_ and σ_fo_ are meridian RMS slope errors of the grating and the pre-mirror, respectively, *W* is the half-width of the ruled area of the grating, and *N* is the number of coherently illuminated grating grooves. The source-limited contribution is no longer relevant here since an FEL source produces transversely coherent radiation. The energy-resolving power is evaluated at two photon energies, 900 eV and 400 eV, where 900 eV is the initial optimized energy value for the *C*_ff_ value as shown in Table 1 ▸. It can be seen from Fig. 2 ▸ that the energy-resolving power of the PGM varies with the different source point positions after *C*_ff_ value compensation. When the grating object distance increases, the energy-resolving power increases, while conversely the energy-resolving power decreases. And the trend of change is consistent for both energy points. This is mainly because the compression ratio of the grating changes with the source point position. When the grating image distance is fixed, a larger grating object distance helps to obtain smaller focused spots at the exit slit, thereby improving the energy-resolving power of the PGM. Although the variation in *C*_ff_ value can also cause a change in energy-resolving power, in this case the function of slight changes in *C*_ff_ is to recover the focusing ability of the VLS grating. The increase or decrease in energy-resolving power is mainly dominated by the grating compression ratio.

## 3. Simulations

To verify the compensation effect, some simulations were conducted to evaluate the energy-resolving power of the PGM. The simulation of the beam propagation is carried out with *MOI* code developed by Shanghai Synchrotron Radiation Facility (Meng *et al.*, 2015 ▸; Meng *et al.*, 2017 ▸; Ren *et al.*, 2019 ▸; Ren *et al.*, 2020 ▸). The model is based on statistical optics for numerical analysis of partial coherent X-rays and has already been applied in the beamline design and analysis of coherent light propagation in both synchrotron (Xue *et al.*, 2018 ▸) and FEL facilities (Xue *et al.*, 2024 ▸) successfully.

**Figure 3:**
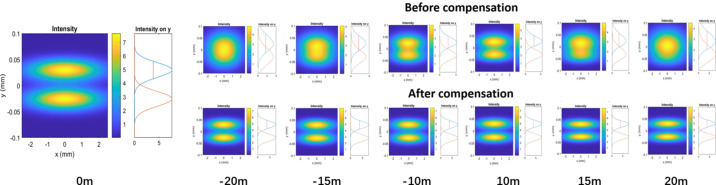
Ray-tracing results of energy-resolving power of the grating monochromator before and after *C*_ff_ value compensation under different source point drifts at 900 eV photon energy.

Fig. 3 ▸ shows ray-tracing results of the energy-resolving power of the grating monochromator before and after *C*_ff_ value compensation under different source point drifts at 900 eV photon energy. In this simulation, several cases including source point drifts of ±10 m, ±15 m and ±20 m are taken as examples and all of the simulations are based on an energy-resolving power of 15000 (*E*/Δ*E*) which is the design value of the grating monochromators.

Even without *C*_ff_ value compensation, the PGM has a certain tolerance for source point drift that the two energies can still be resolved when the source point drifts by ±10 m. However, when the drift of the source point is greater than ±15 m, the two energies can no longer be resolved. Fortunately, the energy-resolving power of the PGM can be recovered with *C*_ff_ value compensation. Even in the case of ±20 m source drift, the two energies can be still resolved well after the *C*_ff_ compensation. Through simulation calculations, it can be seen that the method of adjusting the *C*_ff_ value can effectively compensate for the impact of source point drift on the energy-resolving power of grating monochromators.

## 4. Conclusions

In this work, a method of adjusting the *C*_ff_ value is introduced to compensate for source point drift for soft X-ray beamlines in linear-accelerator-based large-scale FEL facilities. This method can effectively compensate for the impact of source drift on the energy-resolving power of a grating monochromator. The simulation results have demonstrated the fine compensation effect of this method.

## Figures and Tables

**Table 1:** Basic parameters of the grating

Object distance	Imaging distance	Line density	*b*_2_ coefficient	*b*_3_ coefficient	Optimized *C*_ff_
200 m	95 m	200 lines mm^−1^	4.58783 × 10^−5^ mm^−1^	1.56696 × 10^−9^ mm^−2^	1.5 @ 900 eV
